# Supplementary material for: Stable isotope analysis (δ13C and δ15N) of soil nematodes from four feeding groups
Source: PeerJ. 2016 Sep 1;4:e2372. doi: 10.7717/peerj.2372 (PMC5012319; doi:10.7717/peerj.2372)
Supplement: Data S1 [file peerj-04-2372-s001.pdf]

2014 ISO Nematode feeding groups

Well no.	Sample	Description	No. of nem.
1A	14CM-001	*Mononchus*	32
2A	14CM-002	*Rhabditis*	34
3A	14CM-003	*Plectus*	40
4A	14CM-004	*Rotylenchus*	65
5A	14CM-005	*Anatonchus*	3
6A	14CM-006	Aporcelaimidae	20
7A	14CM-007	Qudsianematidae	35
8A	14CM-008	Aporcelaimidae	20
9A	14CM-009	*Rotylenchus*	85
10A	14CM-010	*Mononchus*	22
11A	14CM-011	*Rhabditis*	22
12A	14CM-012	*Plectus*	35
1B	14CM-013	*Rotylenchus*	50
2B	14CM-014	*Mononchus*	17
3B	14CM-015	Aporcelaimidae	18
4B	14CM-016	Qudsianematidae	30
5B	14CM-017	*Rotylenchus*	105
6B	14CM-018	*Plectus*	115
7B	14CM-019	Aporcelaimidae	17
8B	14CM-020	*Mononchus*	30
9B	14CM-021	Aporcelaimidae	25
10B	14CM-022	*Rhabditis*	50
11B	14CM-023	*Rotylenchus*	115
12B	14CM-024	*Plectus*	70
1C	14CM-025	Parasite?	1
2C	14CM-026	Aporcelaimidae	20
3C	14CM-027	*Mononchus*	45
4C	14CM-028	Aporcelaimidae	18
5C	14CM-029	*Rhabditis*	65
6C	14CM-030	*Rotylenchus*	90
7C	14CM-031	*Mononchus*	55
8C	14CM-032	Aporcelaimidae	15
9C	14CM-033	*Plectus*	105
10C	14CM-034	*Rotylenchus*	110
11C	14CM-035	*Rhabditis*	30
12C	14CM-036	*Mononchus*	50
1D	14CM-037	Aporcelaimidae	16
2D	14CM-038	*Rotylenchus*	90
3D	14CM-039	*Rhabditis*	0
4D	14CM-040	*Plectus*	40

Tin cup (mg)	Target weight (mg)	Cup+Nem weight (mg)	Weight of nem (mg)
30.402	30.422	30.422	0.020
29.944	29.964	29.964	0.020
31.983	32.003	32.004	0.021
31.147	31.167	31.169	0.022
31.941	31.961	31.949	0.008
30.929	30.949	30.967	0.038
31.613	31.633	31.636	0.023
30.320	30.340	30.339	0.019
30.682	30.702	30.702	0.020
29.908	29.928	29.935	0.027
31.670	31.690	31.691	0.021
30.660	30.680	30.682	0.022
30.478	30.498	30.500	0.022
31.152	31.172	31.176	0.024
30.717	30.737	30.764	0.047
30.695	30.715	30.708	0.013
31.344	31.364	31.364	0.020
31.158	31.178	31.172	0.014
30.842	30.862	30.865	0.023
30.325	30.345	30.350	0.025
30.358	30.378	30.398	0.040
30.445	30.465	30.468	0.023
29.954	29.974	29.975	0.021
31.301	31.321	31.315	0.014
32.050	32.070	32.071	0.021
30.838	30.858	30.888	0.050
29.984	30.004	30.006	0.022
31.235	31.255	31.282	0.047
31.119	31.139	31.140	0.021
30.760	30.780	30.779	0.019
31.248	31.268	31.270	0.022
30.057	30.077	30.090	0.033
31.732	31.752	31.752	0.020
30.773	30.793	30.793	0.020
30.510	30.530	30.520	0.010
30.305	30.325	30.333	0.028
29.959	29.979	29.983	0.024
31.892	31.912	31.915	0.023
29.972	29.992	29.972	0.000
30.684	30.704	30.701	0.017

Results

Treatment	Rep	Date collected	Amount N (ug)
Organic+FYM	3	05/09/14	1.473
Organic+FYM	3	05/09/14	1.106
Organic+FYM	3	05/09/14	1.568
Organic+FYM	3	05/09/14	1.617
Organic+FYM	3	05/09/14	0.829
Organic+FYM	3	05/09/14	4.529
Organic+FYM	3	05/09/14	1.913
Organic+FYM	3	05/09/14	1.011
Organic+FYM	3	05/09/14	1.562
Organic+FYM	8	05/09/14	1.455
Organic+FYM	8	05/09/14	0.846
Organic+FYM	8	05/09/14	1.476
Organic+FYM	8	05/09/14	1.174
Organic+FYM	8	05/09/14	1.544
Organic+FYM	8	05/09/14	2.641
Organic+FYM	8	05/09/14	1.302
Organic+FYM	8	05/09/14	1.549
Organic+FYM	8	05/09/14	2.124
Organic+FYM	8	05/09/14	2.069
Organic+FYM	9	05/09/14	1.779
Organic+FYM	9	05/09/14	3.534
Organic+FYM	9	05/09/14	2.774
Organic+FYM	9	05/09/14	1.981
Organic+FYM	9	05/09/14	2.008
Organic+FYM	9	05/09/14	1.444
Organic+FYM	9	05/09/14	5.639
Conventional	4	05/09/14	2.572
Conventional	4	05/09/14	5.942
Conventional	4	05/09/14	2.809
Conventional	4	05/09/14	1.998
Conventional	6	05/09/14	2.866
Conventional	6	05/09/14	4.248
Conventional	6	05/09/14	2.349
Conventional	6	05/09/14	1.783
Conventional	6	05/09/14	1.631
Conventional	11	05/09/14	3.876
Conventional	11	05/09/14	2.751
Conventional	11	05/09/14	3.417
Conventional	11	05/09/14	1.148
Conventional	11	05/09/14	2.152

Amount C (ug)	DELTA 13/12C ‰	DELTA 15/14N ‰	Analysed
8.455	9.886	-26.512	Dec-14
6.755	12.124	-26.798	Dec-14
8.334	6.479	-26.208	Dec-14
10.339	2.390	-27.913	Dec-14
5.910	12.784	-27.042	Dec-14
18.241	8.376	-26.011	Dec-14
10.848	10.218	-27.886	Dec-14
6.586	11.154	-26.909	Dec-14
10.857	1.993	-27.936	Dec-14
8.664	12.102	-26.421	Dec-14
6.340	12.144	-27.129	Dec-14
7.996	10.229	-26.296	Dec-14
7.851	3.224	-27.874	Dec-14
8.761	12.644	-26.526	Dec-14
13.009	11.910	-26.721	Dec-14
8.208	12.415	-27.672	Dec-14
10.015	2.489	-28.088	Dec-14
9.872	9.982	-26.033	Dec-14
9.924	10.814	-27.129	Dec-14
10.936	12.791	-26.270	Dec-14
22.624	8.285	-28.322	Dec-14
13.184	9.341	-24.781	Dec-14
13.061	2.034	-28.949	Dec-14
10.221	8.628	-25.123	Dec-14
16.146	6.655	-26.348	Dec-14
28.516	9.751	-28.226	Dec-14
13.819	12.085	-26.485	Dec-14
26.161	8.967	-27.368	Dec-14
13.210	8.568	-25.821	Dec-14
12.774	2.924	-28.670	Dec-14
14.833	10.332	-26.522	Dec-14
20.751	9.432	-27.373	Dec-14
11.407	7.490	-25.906	Dec-14
12.781	1.080	-29.578	Dec-14
4.620	3.278	-23.511	Dec-14
19.343	11.856	-25.509	Dec-14
15.867	8.046	-28.533	Dec-14
16.650	1.396	-28.168	Dec-14
2.382	0.695	-24.304	Dec-14
10.233	8.570	-25.767	Dec-14
